# Cryofibrinogen-associated glomerulonephritis with paraproteinemia

**DOI:** 10.3389/fimmu.2025.1576917

**Published:** 2025-07-15

**Authors:** Xuanli Tang, Mengya Jiang, Huaqin Zhang, Peng Bi, Jun Wang, Tian Ye, Jie Zheng, Mengli Tong, Xingyu Zhu, Xiaotao Hou, Shuhua Bao, Yi Lin, Xue Jiang, Hongyu Chen, Feng Wan, Haichun Yang

**Affiliations:** ^1^ Department of Nephrology, Hangzhou TCM Hospital Affiliated to Zhejiang Chinese Medical University, Hangzhou, China; ^2^ Department of Laboratory, Hangzhou TCM Hospital Affiliated to Zhejiang Chinese Medical University, Hangzhou, China; ^3^ Department of Renal Pathology, King Medical Diagnostics Center, Guangzhou, China; ^4^ Department of Pathology, Microbiology, and Immunology, Vanderbilt University Medical Center, Nashville, TN, United States

**Keywords:** cryofibrinogen-associated glomerulonephritis, membranoproliferative glomerulonephritis, monoclonal gammopathy of renal significance, animal model, cell culture

## Abstract

**Introduction:**

Cryofibrinogen-associated glomerulonephritis (CF-GN) is a rare disease that lacks comprehensive research and requires further investigation to improve our understanding of its pathophysiology.

**Methods:**

Based on the morphological findings from a kidney biopsy and blood tests, an elderly patient was diagnosed with CF-GN. Biological materials obtained from peripheral blood were utilized to treat cultured mesangial cells and mice.

**Results:**

The patient presented with nephrotic syndrome and chronic kidney failure. The biopsy revealed a membranoproliferative glomerulonephritis pattern with distinct substructures and positive fibrinogen staining. Cryofibrinogen was detectable under cold conditions, and monoclonal immunoglobulin (MIg) was exclusively identified within cryoprecipitates. Genetic analysis uncovered an intronic mutation. The patient partially responded to immunosuppressive therapy, but later relapsed with paraproteinemia, and the MIg was detected to have cryoactivity. To investigate the pathophysiology of CF-GN further, its cryoactivity was detected when mixing the serum (with or without MIg) with healthy control plasma. When exposed to the patient’s cryoprecipitates, cultured mesangial cells showed significant proliferation, phagocytosis of fibrinogen, and lysosomal degeneration. Injection of these cryoprecipitates into mice induced proliferative glomerulonephritis and other organ damage.

**Conclusion:**

This study provides valuable insights into the diagnosis, treatment, and pathophysiology of CF-GN with paraproteinemia. The identification of the complex of cryofibrinogen and MIg as a potential mechanism of glomerular damage shed light on the pathogenesis of this rare disease.

## Introduction

Cryofibrinogenemia (CF) is a unique medical condition characterized by the presence of cryoprotein in plasma precipitates when cooled (1, 2). Cryofibrinogenemia may be primary (essential) or secondary to other underlying disorders, such as carcinoma, infection, vasculitis, autoimmune disease, or associated with cryoglobulinemia (3–7). Main clinical manifestations mostly involve the skin, whereas renal involvement is rare (4% to 21%) (8–12). The pathological characteristics included a membranoproliferative glomerulonephritis (MPGN) pattern, positive fibrinogen staining with negative or weak staining for immunoglobulins (Igs) and complements, and large microtubular structures under electron microscopy (EM) (13). These features helped distinguish the condition from cryoglobulinemic glomerulonephritis (CryoGN). In elderly patients, some case reports have identified paraproteinemia as the second cause and the primary culprit in the development of CF-GN (11, 14). However, the mechanisms underlying the development of cryofibrinogen-associated glomerulonephritis (CF-GN) and the role of monoclonal immunoglobulin (MIg) in cryoprecipitate formation and subsequent kidney injury remain largely unexplored, and there are no established diagnostic criteria or consensus regarding optimal management (12).

This study aimed to analyze the clinicopathological characteristics, treatment, and outcomes of persistent cryofibrinogenemia associated with paraproteinemia. Furthermore, to explore the etiology and pathogenesis of CF-GN, an animal model of systematic injury via CF injection was established, and CF-induced mesangial cell injury stimulated by CF and MIg was analyzed *in vitro*. Additionally, the relationship between MIg and CF was hypothesized and clarified using cryoactivity experiments.

## Methods

### Patient and animals

A 73-year-old female was admitted with mild edema and abnormal urinalysis. Cryoprecipitates from her plasma were detected and collected to create the animal model. Eight male C57BL/6 mice, 6–8 weeks old and weighing 16–24 g, were purchased from Shanghai Sippe-Bk Lab Animal Corporation. Written informed consent was obtained from the patient for the publication of any potentially identifiable images or data included in this article. All clinical and experimental protocols were approved by the ethics committees of Hangzhou TCM Hospital Affiliated to Zhejiang Chinese Medical University (2023KLL081) and Zhejiang Chinese Medical University (IACUC-20230227-09).

### Pathology detection in renal tissue

The frozen and paraffin-embedded samples were stained by immunofluorescence (IF) including fluorescein isothiocyanate (FITC) conjugated IgA, IgG, IgM, C3c, C1q, κ, λ, and polyclonal rabbit anti-human fibrinogen antibody (F0111, 1:50, DAKO, Denmark). Monoclonal mouse anti-human CD68 (Kit-0026, Maxim Corporation, China) was detected by immunohistochemistry. The remaining paraffin-embedded tissue was microdissected and analyzed by mass spectrometry (LMD-MS). EM and immunoelectron microscopy (IEM) to detect fibrinogen, γ (SAB3701290, 1:100, Sigma), κ, and λ were performed by standard protocols and were observed under a transmission electron microscope (JOEL-1400, Japan). Details are provided in **Supplementary Materials** and **Methods**.

### Detection of cryoprecipitates

The patient’s blood was collected into a pre-warmed tube without anticoagulant for cryoglobulin detection and into an EDTA anti-coagulant tube for cryofibrinogen detection. Both the sera and plasma were centrifuged at 37°C and then cooled to 4°C for 7 days. Precipitates were observed at 4°C and dissolved by warming to 37°C. The concentration of fibrinogen in the plasma and precipitates was measured by the von Clauss method on a coagulation analyzer (Sysmex, Japan).

Monoclonal immunoglobulins, including IgA, IgG, IgM, κ, λ, and fibrinogen in serum, plasma, and precipitates were detected by immunofixation electrophoresis (IFE) on a Hydrasys device (Sebia, Evry, France) according to the manufacturer’s protocols. Free light chains were detected by FreeliteTM at the UK Binging Site on a Behring BNII fully automatic apparatus.

The precipitates were centrifuged, embedded into 2.5% agarose, and fixed into 2.5% glutaraldehyde overnight. The approach for both EM and IEM were performed as above.

### Genetic analysis

A specialized gene panel targeting thrombosis and hemostasis-related genes, including fibrinogen, were sequenced using next-generation sequencing (NGS) techniques, with DNA capture facilitated by a GenCap capture kit (MyGenostics Inc, Beijing, China). Bioinformatics analyses were conducted using standard software. All mutations identified by DNBSEQ-T7 sequencing and genomic DNA from the available family members were verified through by Sanger sequencing.

PCR amplification was performed using FGB-specific exon-spanning primers (spanning exons 1–7). The PCR products were then separated on a 1% agarose gel and visualized using a gel imaging system. Total RNA was extracted and cDNA was synthesized using a Primescript™ RT reagent kit (TaKaRa, Kusatsu, Japan). The quantitative analysis of fibrinogen alpha chain (FGA), fibrinogen beta chain (FGB), and fibrinogen gamma chain (FGG) mRNA was performed using qRT-PCR on an Applied Biosystems QuantStudio 5 Real-Time PCR System. Details are provided in **Supplementary Materials** and **Methods**.

### Cryoactivity of MIg

To study the interaction between MIg and cryofibrinogen, we first incubated the patient’s serum from the first admission (no MIg) and the second (with MIg) with plasma from healthy controls at 4°C, as previously described (14). A serum with IgG λ paraprotein from a patient with monoclonal gammopathy of unknown significance (MGUS) was used as a negative control. Next, we removed IgG λ using Protein A beads (Pierce™) and applied the supernatant to a gravity column. Furthermore, molecular docking was applied to predict whether there are interaction sites between fibrinogen and MIg.

### Animal model

Four mice received monthly intraperitoneal injections of cryoprecipitates from the patient with high-dose (two mice with 300 μl) and low-dose (two mice with 200 μl), and four mice were controls. Two low-dose mice were sacrificed after the third and fourth injections, while high-dose mice were sacrificed after the final injection. The urinary protein-to-creatinine ratio (UPCR) was monitored monthly, and serum ALB and Scr levels were tested pre-injection and at sacrifice. At the end of the experiment, the mice were anesthetized via intramuscular injection of 50 mg/kg Zoletil, followed by cardiac blood sampling and collection of tissue samples. Euthanasia was then performed by cervical dislocation. Tissues were processed for light microscopy (LM) and EM. Kidney sections were stained for fibrinogen, Alexa Fluor (AF) 594-conjugated goat anti-mouse IgG (ab150116, 1:100, Abcam, UK), AF647-conjugated goat anti-mouse IgM (ab150123, 1:100, Abcam, UK), and FITC-conjugated goat anti-mouse IgA (ab97234, 1:100, Abcam, UK) by IF method, and rat anti-mouse C5b-9 (sc-66190, 1:50, Santa Cruz, USA) by IHC method. Other organs including brain, lung, heart, liver, intestine, and skin were processed and examined by LM and EM. Details are provided in **Supplementary Materials** and **Methods**. All authors complied with the ARRIVE guidelines during animal experiments.

### Cell culture

Human mesangial cells (HMCs) were kindly gifted by Prof. Chunhua Weng of Zhejiang University, Medical College (Hangzhou, China). To further investigate the role of cryoprecipitates and MIg in mesangial cells, HMCs were cultured in DMEM (Gibco, Grand Island, NY) and treated with cryoprecipitates and purified MIg (IgG λ from the patient and other individuals) for 24h to examine cell proliferation, fibrinogen/MIg uptake, and ultrastructure. Cell proliferation was assessed using a BeyoClickTM 594 EdU kit (Beyotime, Shanghai, China) and quantified with Harmony 4.8 imaging and analysis software (PerkinElmer). Fibrinogen and IgG λ uptake was detected by IF and visualized with Cytospin 4 (Thermo Fisher Scientific). EM was performed as previously described for cryoprecipitates. Details are provided in **Supplementary Materials** and **Methods**.

### Statistical analysis

Cell counts and mesangial matrix area were quantified using Image-Pro Plus 6.0, and the average values were analyzed with GraphPad Prism (version 6.01).

## Results

### Clinical history

A 73-year-old woman with mild edema and abnormal renal function was admitted with a history of hypertension for more than three years (up to 180/90 mm Hg) and a cerebral infarction six months ago. Laboratory tests revealed increased serum creatinine (Scr, 126 mmol/L), proteinuria (3.4 g per 24h), and hematuria (7–8 red blood cells/ul). Serum albumin was 28.7 g/L, hemoglobin was 95 g/L, and complement C3 was 76 mg/dL. Coagulation markers and serological tests for antinuclear antibodies, antineutrophil cytoplasmic antibodies, MIg, and malignancy were normal. Physical examination showed mild pitting edema of the lower extremities, with no rash, joint swelling, or abdominal tenderness. She was taking nifedipine, irbesartan, aspirin, and atorvastatin. The patient was clinically diagnosed with nephrotic syndrome and chronic kidney disease stage 3.

**Figure 1:**
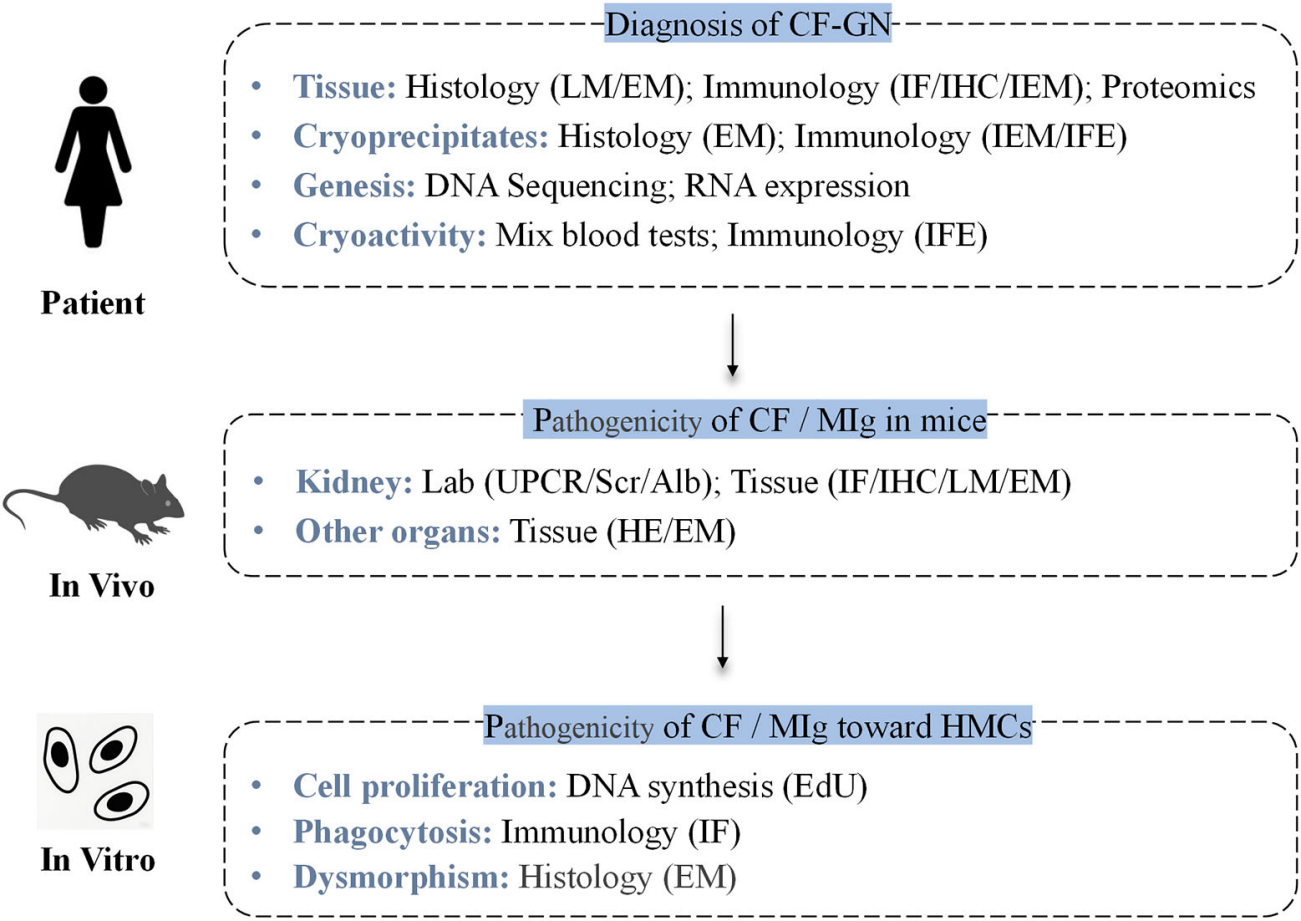
Flowchart of the experimental procedures, including patient studies, *in vivo*, and *in vitro* experiments. CF-GN, cryofibrinogen-associated glomerulonephritis; CF, cryofibrinogenemia; MIg, monoclonal immunoglobulin; HMCs, human mesangial cells; IF, immunofluorescence; IHC, immunohistochemistry; LM, light microscopy; HE, hematoxylin & Eosin Staining; EM, electron microscopy; IEM, immunoelectron microscopy; MS, mass spectrometry; IFE, immunofixation electrophoresis.

The flowchart of the experimental procedures is shown in **Figure 1**.

### Kidney biopsy

**Figure 2:**
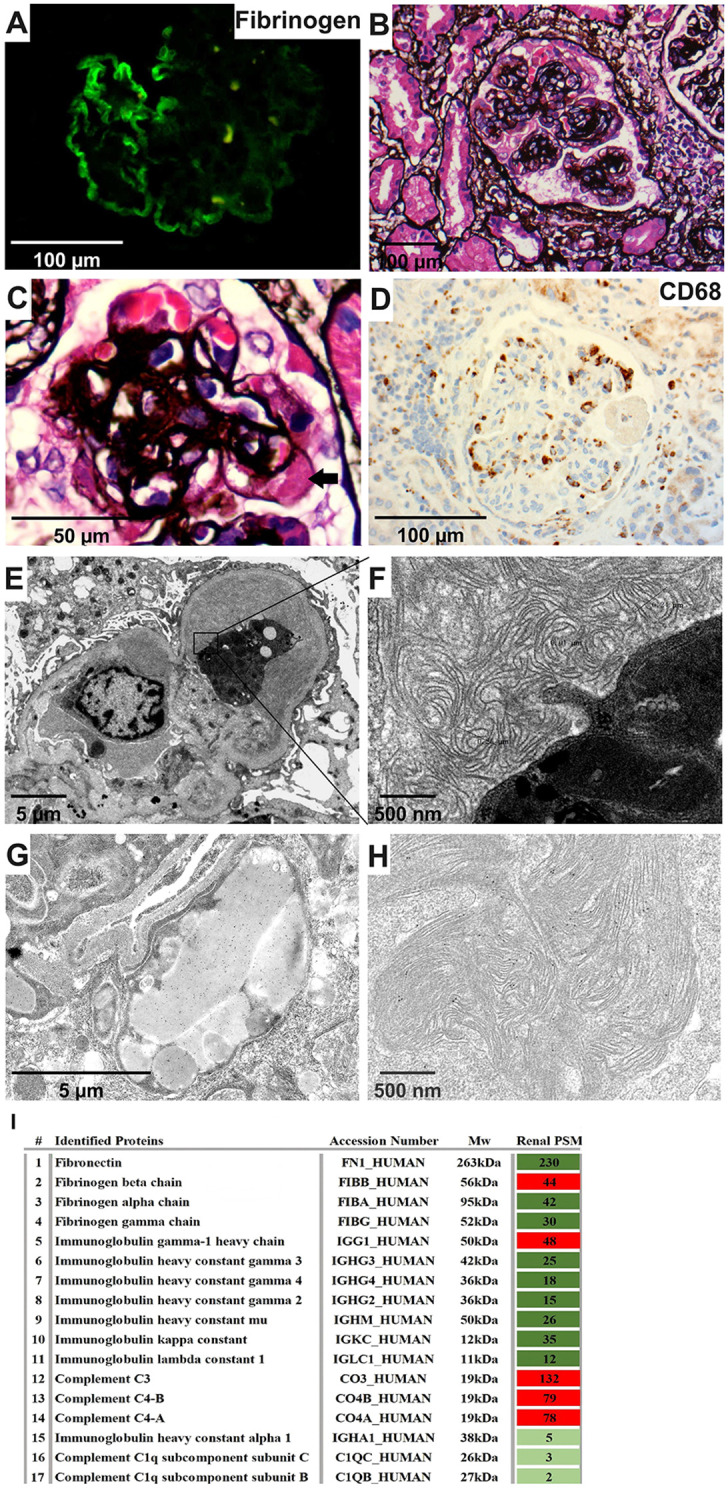
Renal pathology characteristics of the patient. **(A)** Fibrinogen was positive along capillaries (IF, 400×). **(B, C)** Diffuse mesangial expansion, nodular sclerosis, and a hyaline thrombi (arrow) (PASM, 400×&1000×). **(D)** CD68 was enriched within capillaries (IHC, 400×). **(E, F)** A thrombi with large tubules and curved fibril substructures surrounded by macrophages (EM, 4000×&40000×). **(G, H)** Gold-labeled fibrinogen was intensively distributed in lysosomes of the macrophage within the capillary lumen and was also scattered in the substructure of the mesengial area (IEM, 10000×&40000×). **(I)** Proteomic analysis of kidney tissue showed the highest proportion of the fibrinogen beta chain, together with relatively high spectral counts of IgG1, C3 and C4 (MS). IF, immunofluorescence; PASM, periodic Schiff-methenamine staining; IHC, immunohistochemistry; EM, electron microscopy; IEM, immunoelectron microscopy; MS, mass spectrometry.

Immunofluorescence (IF) showed segmental IgM and C3 deposits in the glomeruli, with negative results for IgA, IgG, C1q, κ, and λ. Fibrinogen staining by IF revealed a subendothelial “sausage-like” pattern (**Figure 2A**). Light microscopy (LM) displayed a MPGN pattern and nodular sclerosis (**Figures 2B, C**). There was a massive infiltration of CD68+ macrophages in the glomerular tuft (**Figure 2D**). Electron microscopy (EM) further elucidated diffuse foot process effacement and dense deposits within the mesangial and subendothelial areas, many of which exhibited substructures. Scattered thrombi with large tubules (250–350 nm in diameter) and curved fibrils with random distribution (10 nm in diameter), surrounded by macrophages, were detected in the capillary lumen (**Figures 2E, F**). Immunoelectron microscopy (IEM) confirmed fibrinogen deposition in mesangial and thrombi substructures, as well as in macrophage lysosomes, while γ, κ, and λ were negative (**Figures 2G, H**).

Mass spectrometry of renal tissue identified high levels of fibronectin and fibrinogen, predominantly the fibrinogen beta chain. IgG1, C3 and C4 levels were also elevated, in contrast to lower immunoglobulin (Ig) and light chain levels (**Figure 2I**). Therefore, the pathology diagnosis was cryofibrinogen-associated glomerulonephritis (CF-GN).

### Cryoprecipitates analysis and genetic test

**Figure 3:**
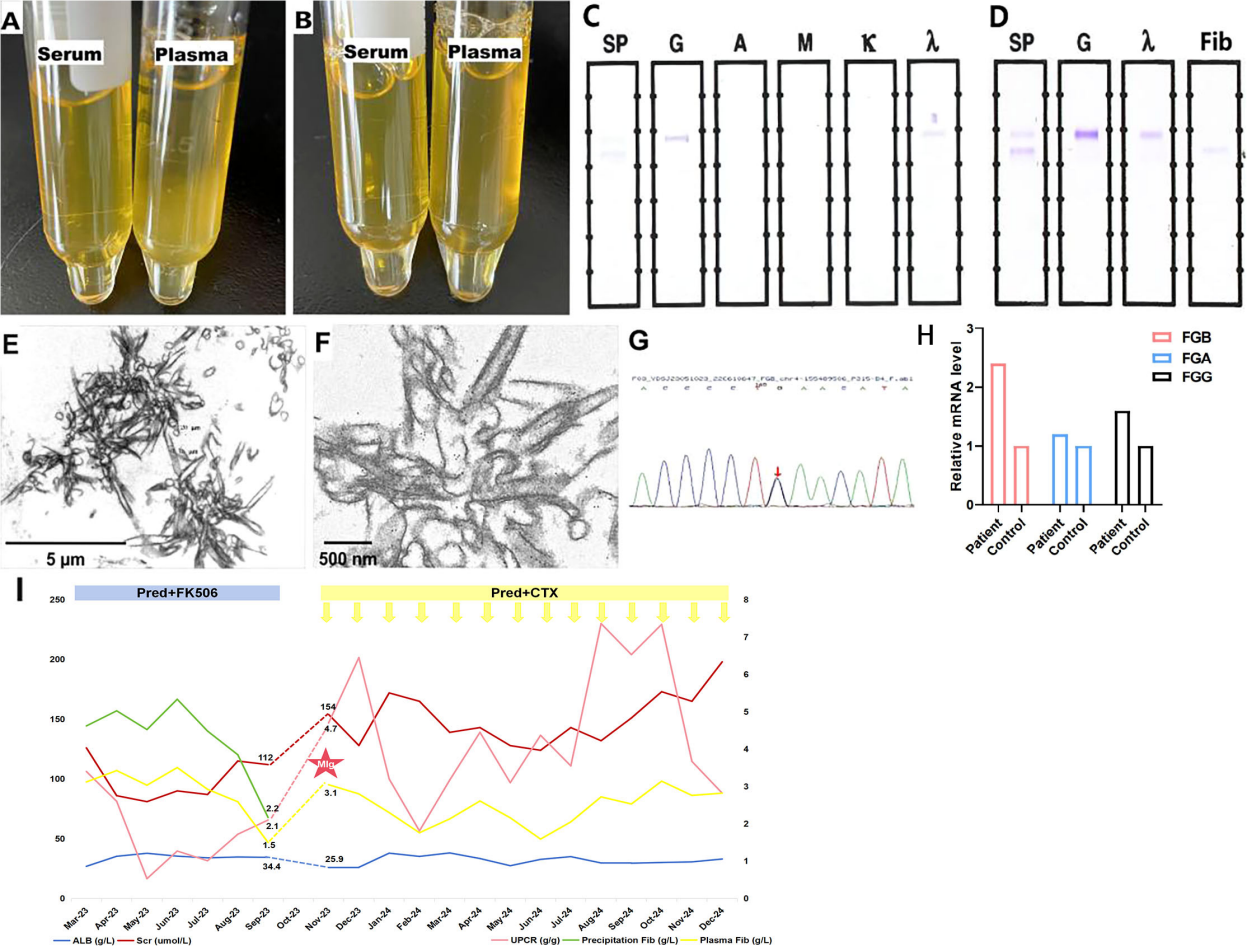
Cryoprecipitates detection. **(A)** The precipitates were detected in patient’s plasma but not in serum under 4°C. **(B)** The precipitates dissolved under 37°C. **(C, D)** Monoclonal IgG λ was detected in precipitates coexisting with weak fibrinogen (IFE). **(E)** Crystal-like precipitates with straight column shape at vertical section and tubules with spiral shape at cross-section (10000×). **(F)** Gold labeled fibrinogen scattered within the crystal area (IEM, 40000×). **(G)** Genetic analysis identifed heterozygous c.719-27G>C variant in intron 4 of *FGB* gene. **(H)** The relative mRNA expression level of *FGB* was more than two times higher in the patient comparing with her daughter, followed by *FGG* and *FGA*. **(I)** Treatment and follow-up of fibrinogen level and kidney injury. *FGB*: fibrinogen beta chain; *FGG*, fibrinogen gamma chain; *FGA*, fibrinogen alpha chain; Scr, serum creatinine; ALB, albumin; CTX, cyclophosphamide; Pred, prednisone; UPCR, urinary protein-to-creatinine ratio; Fib, fibrinogen, MIg, monoclonal immunoglobulin.

Cryoprecipitates were observed in plasma but not in serum at 4°C and dissolved upon warming to 37°C (**Figures 3A, B**). Although serum and plasma IFE tests were negative, IFE of the cryoprecipitates identified IgG λ along with a faint fibrinogen band, indicating the coexistence of cryofibrinogen with MIg (**Figures 3C, D**). Fibrinogen levels were 3.1 g/L (normal 2–4 g/L) in plasma and 4.6 g/L in the precipitates. EM of the cryoprecipitates showed crystal structures similar to those seen in kidney biopsy deposits, and IEM revealed sparse gold-labeled fibrinogen, correlating with the kidney findings (**Figures 3E, F**).

Genetic analysis revealed a heterozygous c.719-27G>C variant in intron 4 near the splice site of the *FGB* gene in the patient (**Figure 3G**). This mutation was also present in the patient’s son, but not in her daughter. Although predicted as a variant of uncertain significance, the Bayesian framework score indicated a pathogenicity probability of 18.8% (14). For further investigation, we performed PCR to examine the length of FGB cDNA to determine whether the intron mutation affects the splicing site. However, the PCR product bands of both the healthy control and the patient were 1156 bp in size, suggesting that the intron 4 mutation does not affect FGB splicing. In addition, qRT-PCR was used to measure the mRNA levels of fibrinogen alpha chain (FGA), fibrinogen beta chain (FGB), and fibrinogen gamma chain (FGG). The patient’s FGB level was 2.4 times higher than that of her daughter, while FGG was 1.6 times higher, and FGA was 1.2 times higher (**Figure 3H**).

### Follow-up

The patient was treated with oral prednisone (1 mg/kg per day, tapered) and FK506 (1 mg/kg, orally, once daily). Scr and urine protein-to-creatinine ratio initially decreased, but increased again three months later. Nephrotic syndrome recurred after another three months due to a lack of FK506, resulting in hospital readmission. Plasma fibrinogen levels returned to baseline and serum C3 decreased significantly (66.5 mg/dL). IgG λ MIg was detected by IFE in both precipitates and serum. Bone marrow biopsy and flow cytometry showed 0.5% mature plasma cells without light chain restriction, and the serum free light chain ratio was normal (0.76, normal 0.31-1.56). No other cryofibrinogen-associated systemic disease was found. Therefore, the patient was diagnosed with MGUS. Treatment was adjusted to corticosteroids with cyclophosphamide (0.6g per dose, intravenous infusion), resulting in a stable renal and hematologic conditions over an additional one year of follow-up (**Figure 3I**).

### MIg-Fibrinogen interaction in cryoprecipitation

**Figure 4:**
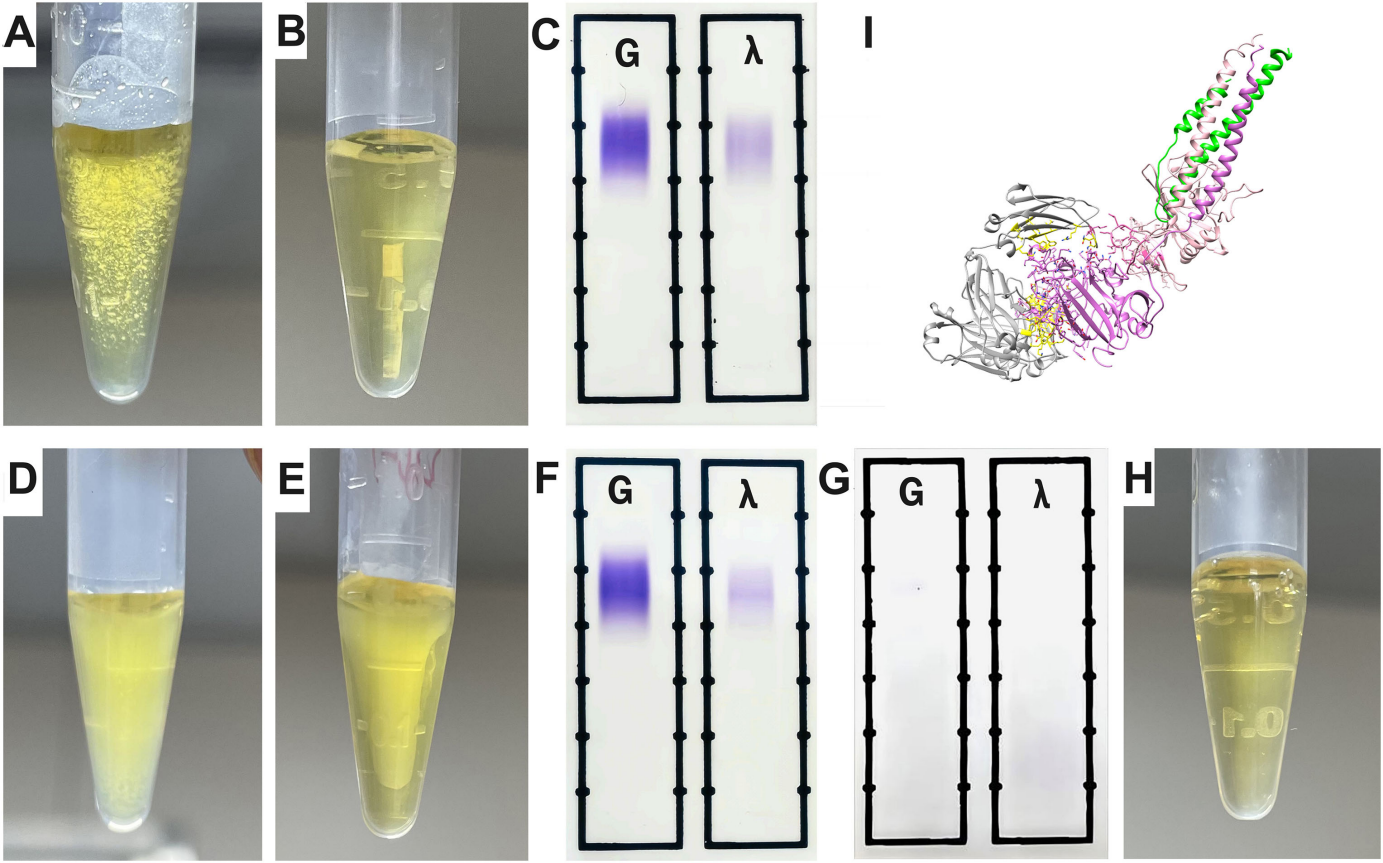
The interaction of MIg with fibrinogen. **(A)** The patient’s serum (first admission without MIg) mixed with plasma from a health control showed granular cyroprecipitates under 4°C. **(B)** Wholly dissolved mixed blood of panel **(A)** under 37°C. **(C)** IFE detection of the mixed blood from panel B showing negative MIg. **(D)** Serum of the patient (second admission with MIg) mixed with plasma from a health control showed gelatinous cyroprecipitates under 4°C. **(E)** Partially dissolved mixed blood of panel D under 37°C. **(F)** IFE detection of blood in panel **(E)** showed MIg (IgG λ). **(G)** IFE was negative when IgG including MIg in the serum of her second admission was extracted. **(H)** The patient’s serum after extraction was mixed with plasma from a health control, and no cryoprecipitates were detected under 4°C. **(I)** The molecular docking revealed a robust interaction between MIg and fibrinogen. Grey, IgG Fab fragment; Purple, fibrinogen alpha chain; Yellow, the residues involved in the interface; Green, fibrinogen beta chain; Light pink, fibrinogen gamma chain. IFE, immunofixation electrophoresis; MIg, monoclonal immunoglobulin.

To further investigate cryofibrinogen formation in this patient, we mixed her serum with plasma from a healthy control. The patient’s serum from the first admission, which did not show MIg, formed granular cryoprecipitates at 4°C and completely dissolved at 37°C (**Figures 4A–C**). In addition, serum from the second admission, which showed MIg, formed gelatinous cryoprecipitates at 4°C and only partially dissolved at 37°C, whereas the MIg from other individuals couldn’t form cryoprecipitates (**Figures 4D–F**). These observations suggest that MIg may be critical for cryofibrinogen formation. Next, we removed IgG from the serum of the second admission, and the remaining serum components mixed with normal plasma did not induce cryoprecipitates (**Figures 4G, H**). Molecular docking revealed the extra-molecular interactions between fibrinogen and IgG Fab fragment, with a total of 3012 Ångströms^2^ buried in the interface, indicating a close contact between these molecules (**Figure 4I**).

### Mice with cryoprecipitate injection

**Figure 5:**
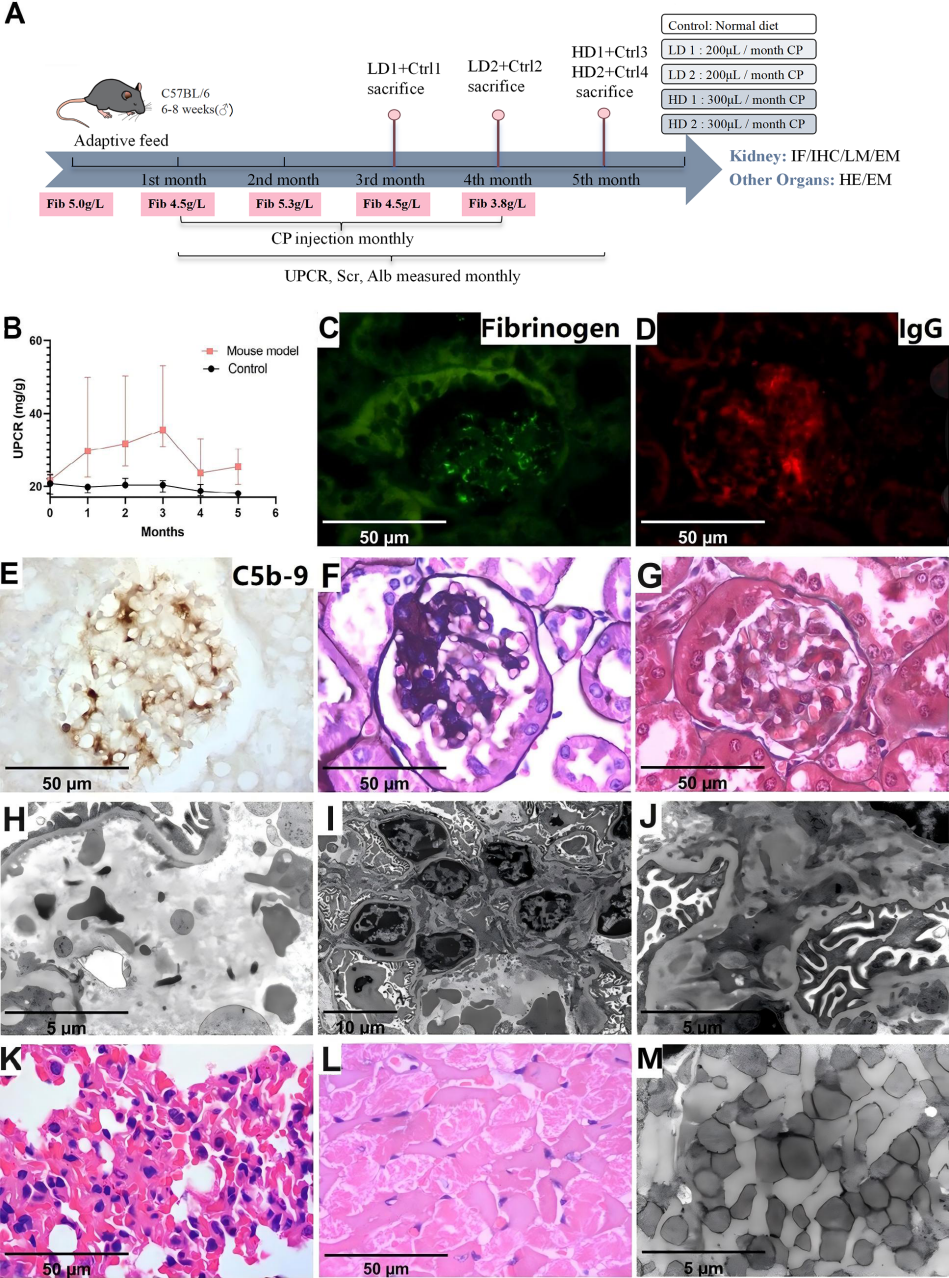
Laboratory data and pathological characteristics in animal model injected with cryoprecipitates. **(A)** The animal model experimental workflow. **(B)** Laboratory data showed significantly increased proteinuria in the mouse model compared to the control (P<0.05). **(C)** Positive fibrinogen within the capillary lumen (IF, 1000×). **(D)** Positive IgG in mesangial area (IF, 1000×). **(E)** Positive C5b-9 in mesangial area (IHC, 1000×). **(F)** Mesangial proliferation (PASM, 1000×) **(G)** Immune complex deposits in mesangial area (Masson, 1000×). **(H)** Scattered fibrin tactoid (EM, 10000×). **(I)** Mesangial expansion and cell proliferation (EM, 3000×). **(J)** Electron dense deposits in mesangial area, but without substructure (EM, 10000×). **(K)** Intra-alveolar red cell extravasation and neutrophil infiltration (HE, 1000×). **(L)** Myocardial necrosis (HE, 1000×). **(M)** Loss of striated muscle fibers (EM, 10000×). LD, low dose; HD, high dose; Ctrl, Control; Fib, fibrinogen; CP, cryoprecipitates; UPCR, urinary protein-to-creatinine ratio; Scr, serum creatinine; ALB, albumin; IF, immunofluorescence; IHC, immunohistochemistry; LM, light microscopy; PASM, periodic Schiff-methenamine staining; EM, electron microscopy.

To investigate the effect of cryofibrinogen on glomeruli *in vivo*, we injected mice with cryofibrinogen or vehicle. The animal model experimental workflow is depicted in **Figure 5A**. Mice receiving cryofibrinogen injections showed significantly increased proteinuria compared to the vehicle group (*P*=0.0045) (**Figure 5B**). However, it peaked and began to decrease after three injection cycles, suggesting a robust innate immune response in mice. Serum creatinine and serum albumin levels had no significant difference between the two groups (*P*>0.05). Kidneys harvested at sacrifice showed positive fibrinogen in the glomerular capillary lumen, while IgG and C5b-9 were positive in the mesangial area (**Figures 5C–E**). LM showed significant mesangial expansion and immune deposits (P<0.001) (**Figures 5F, G**). EM showed fibrin tactoids within the capillary lumen, mesangial proliferation and mesangial electron dense deposits without substructures (**Figures 5H, J**). These findings suggest the development of immune complex-mediated mesangial proliferative glomerulonephritis (IC-MesPGN) in cryoprecipitate-injected mice.

Skin, brain, liver and intestine showed normal morphology in both groups. Two mice that received cryoprecipitates injections showed intra-alveolar red cell extravasation and neutrophil infiltration in the lungs, while one mouse exhibited focal necrosis by LM and loss of striated muscle fibers by EM of the heart (**Figures 5K–M**). However, the coronary artery and aorta were not occluded.

### Effect of cryoprecipitates and MIg on mesangial cells

**Figure 6:**
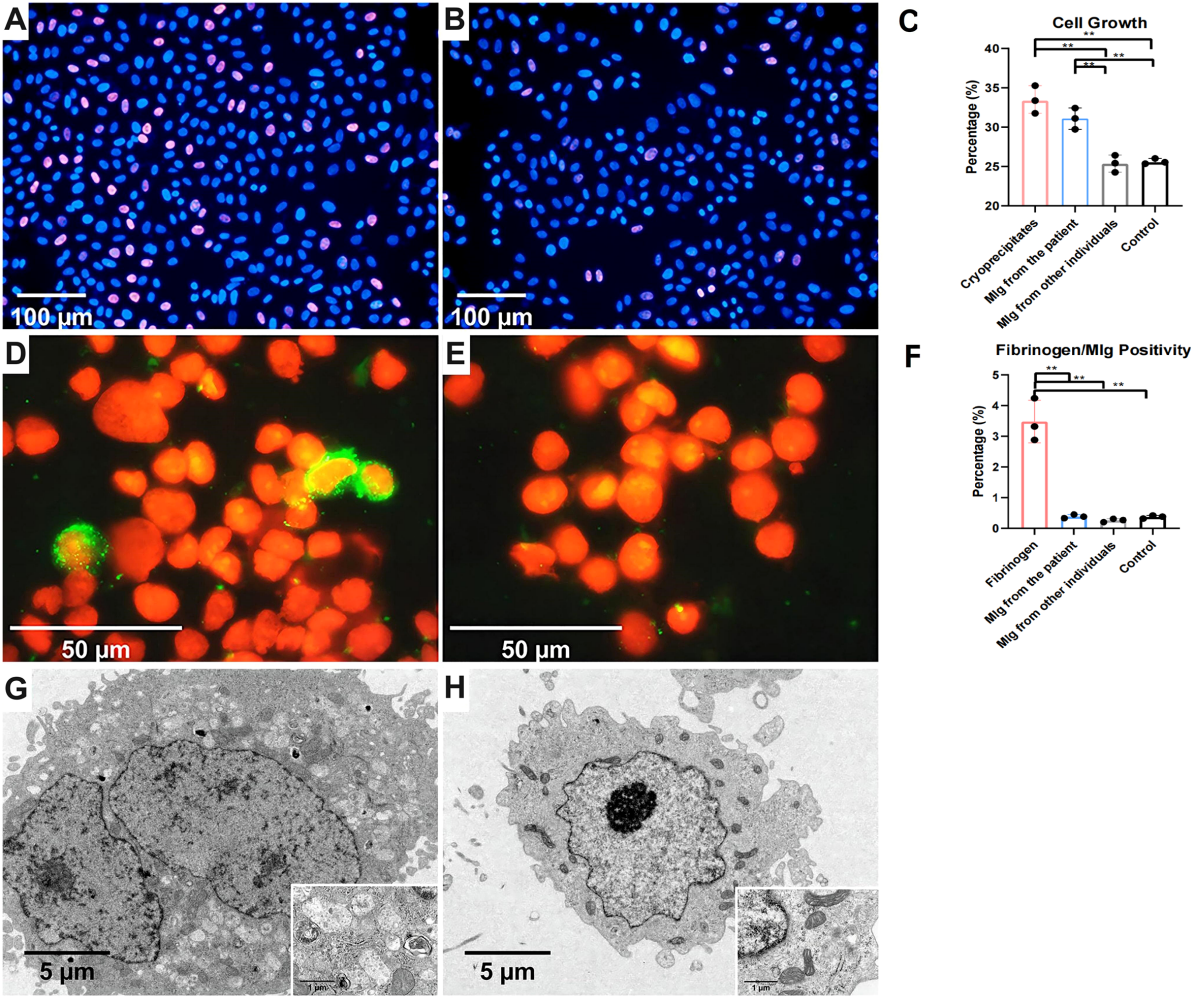
Human mesangial cells stimulated by cryoprecipitates. **(A)** Higher proportion of EdU positive cells (purple red) in the cryoprecipitates’ group than the control group **(B)** (IF, 200×). **(C)** The proliferation of mesangial cells induced by cryoprecipitates and MIg from the patient was statistically higher than that induced by MIg from other individuals and the vehicle control (P<0.01). **(D)** Fibrinogen positive HMCs (green) in the precipitates group but not in the control group **(E)** (IF, 1000×). **(F)** Cryoprecipitate-induced fibrinogen positive HMCs significantly outnumbered those induced by MIg or vehicle (P<0.01). **(G)** Mesangial cell hypertrophy, mitochondrial depletion and lysosomal degeneration in the cryoprecipitate and MIg from the patient groups, but not in other groups **(H)** (EM, 5000× of large images & 30000× of inserted images). **P < 0.01.

To investigate the direct effects of cryoprecipitates and MIg on mesangial cells, we cultured mesangial cells with cryoprecipitates, MIg and vehicle control. The percentage of EdU-positive cells in the cryoprecipitates and MIg from the patient was significantly higher compared to that in MIg from other individuals and in the vehicle control, indicating that the patient’s cryoprecipitates and MIg stimulate mesangial cell proliferation (**Figures 6A–C**). In addition, the proportion of fibrinogen-positive cells increased in the cryoprecipitates group, suggesting cytophagic activation (**Figures 6D–F**). In contrast, the uptake of MIg was negligible, whether from the patient or from other individuals. EM revealed that approximately 50% of mesangial cells in the cryoprecipitates and MIg from the patient group exhibited hypertrophy, mitochondrial depletion, and lysosomal degeneration, whereas less than 10% of cells in the MIg from other individuals and the vehicle control group showed this phenotype (**Figures 6G, H**). However, no unique substructures or fibrin tactoids were observed in the cells.

## Discussion

In CF patients, laboratory analyses commonly show normal levels of fibrinogen and complement, with a few exceptions exhibiting increased levels and prolonged D-dimers (2, 3, 9, 11, 14–17). In our case, fibrinogen levels in plasma remained within the normal range throughout the entire course of the disease, and C3 level was slightly decreased at onset but became conspicuously lower upon the relapse of NS, indicating immune system activation. The histological features of CF-GN present predominantly as an MPGN pattern, yet exhibit limited Ig and complements deposition, which might be easily misdiagnosed as cryoglobulinemic glomerulonephritis (CryoGN), chronic thrombotic microangiopathy (TMA), C3 glomerulonephritis, or fibronectin glomerulopathy (3, 4, 6, 11, 17–21). Therefore, fibrinogen staining on renal tissue is crucial when these histological characteristics are present. In this patient, fibrinogen deposition was predominantly observed in the subendothelial area, demonstrating a characteristic “sausage-like” pattern that corresponded to electron-dense deposits on EM, which is more frequently observed in immune-complex-mediated MPGN. Clinically, skin manifestations often serve as the initial indicators and arterial or venous thrombotic events are frequent (9, 22–26). The occurrence of kidney disease is relatively rare, ranging from 0-22% (8, 9, 18, 22, 27, 28). Despite this, 74% IgA nephropathy was reported to show positive CF in plasma, even though no clear association between CF and IgA nephropathy has been illustrated (6). Clinical manifestations of CF-GN are variable, including 10% with a positive MIg (4, 6, 12, 29, 30). In our study, the patient manifested kidney-dominant injury but without dermatological impairment, which may be related to the specific physicochemical properties of cryoprecipitates. In the animal study, we found that the heart and lungs were involved, in addition to kidney injury, indicating multi-organ injury and the variety of clinical manifestations seen in patients.

Some case reports have described paraproteinemia in CF-GN patients (11, 14, 18–20, 31, 32). The monoclonal component may exist in serum but not in cryoprecipitates or only appear after cryoprecipitation (11, 14, 17, 18). However, the pathogenesis of MIg and its association with CF remain elusive. Euler HH et al. postulated that the MIg with cryoactivity acted as an antibody against fibrinogen and caused cryoprecipitation, whereas Gant CM et al. hypothesized that cryoprecipitate formation was directly related to the presence of MIg (14, 16). Nash JW et al. suggested that MGUS might be a more important cause of cryofibrinogenemia than previously thought (18). In our case, the serum with MIg of the patient had cryoactivity, even though it was undetected by IFE on her first admission; whereas the serum without MIg or the serum with monoclonal protein from other patients did not exhibit cryoactivity. Furthermore, the molecular docking indicates that fibrinogen might have a close contact with IgG Fab fragment. Those phenomena suggest that the patient’s MIg has special characteristics that induce fibrinogen with genetic variants to form cryoprecipitates, which might be the culprit of CF, even in trace quantities.

Some studies suggest that CF has a familial inheritance with an autosomal dominant mode (25, 28, 33–35). Nevertheless, it was insufficient to confirm without a genetic test. In our case, a heterozygous intronic mutation in the FGB gene was identified in both the patient and her son, which might not affect the conformation of fibrinogen protein, but appeared to relatively increase the mRNA expression level of FGB in the patient. Moreover, LMD-MS identified the highest proportion of FGB protein among all chains. Therefore, we performed experiments including PCR and RT-PCR to determine whether the intron mutation affects cDNA length and mRNA level. The results demonstrated that the intron mutation did not alter the cDNA length of FGB but increased its mRNA expression. We hypothesize that intronic mutations may increase expression levels by enhancing transcription, relieving repression, stabilizing mRNA, or altering epigenetic states (36). Increased mRNA expression may elevate beta protein levels or alter the characteristics of fibrinogen, potentially increasing its affinity for MIg and causing abnormal fibrinogen consumption. This genetic background likely serves as the first hit predisposing to CF formation. Crucially, the presence of isomeric MIg acts as a second hit by binding fibrinogen and forming a mega-complex that precipitates under cold conditions. This pathogenesis resembles that of complement mediated thrombotic microangiopathy, potentially explaining the disease’s rarity. The patient developed symptoms late, possibly due to gradual MIg accumulation or MPGN’s slow progression. Her son carried the same genetic variant but remained asymptomatic due to the absence of paraprotein, confirming that MIg acts as an essential “second hit” in disease pathogenesis.

Hatzfeld JA et al. reported that fibrinogen stimulates the proliferation of human hematopoietic cells *in vitro* (7). Our *in vitro* study revealed mesangial cell proliferation, abnormal morphological features, and phagocytosis of fibrinogen in the cryoprecipitates group, suggesting that the toxicity of cryoprecipitates may lead to MPGN lesions *in vivo*. In addition, mesangial cells exhibited significant proliferation and dysmorphism in response to patient-derived MIg, but not to MIg from other individuals, underscoring the pivotal role of patient-specific MIg in the pathogenesis of cryoprecipitates. As previously reported, MIg can induce proliferative glomerulonephritis (e.g., CryoGN and proliferative glomerulonephritis with monoclonal immunoglobulin deposits), which could explain the proliferation and dysmorphic features of HMCs after stimulation (37, 38). However, the HMCs internalized only the cryoprecipitates but not the MIg alone. The results indicate that the fibrinogen-MIg cryoprecipitate complex, but not MIg alone, can functionally induce mesangial cell transformation and lead to persistent tissue injury.

**Figure 7:**
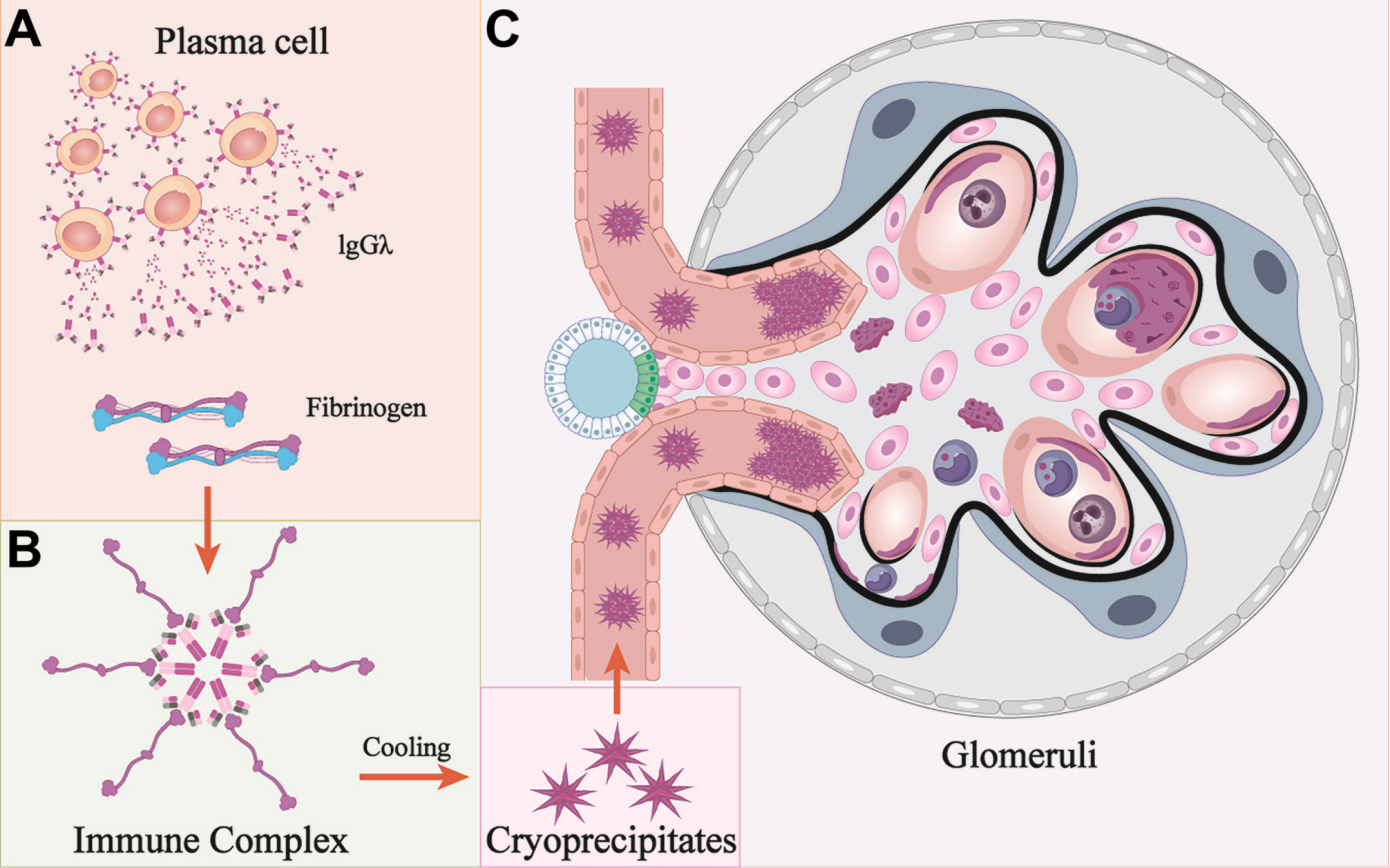
Mechanisms of cryofibrinogen-associated glomerulonephritis. **(A, B)** Monoclonal protein (IgG λ) is secreted from a cluster of plasma cells, and interact with fibrinogen to form immune complex. **(C)** Cryoprecipitates form under cooling conditions and deposit at multiple locations. These deposits are engulfed by macrophages and cause membranoproliferative glomerulonephritis. Pink cells: mesangial cells; Blue cells: podocytes; Dark blue cells: macrophages; Grey cells: neutrophils; Black curved lines: GBM.

Besides, the animal model of proliferative injuries was similar to those in patients after continuous injection of cryoprecipitates. Although we injected mice with varying doses of cryoprecipitates, neither the observed proteinuria nor the morphological changes demonstrated dose dependency. This suggests that the pathological effects may be initiated upon reaching a threshold concentration of pathogenic factors rather than through cumulative dose effects. Pathologically, the presence of IgG and the absence of substructures in the mouse model suggests that a polyclonal IgG complexed with human fibrinogen is morphologically distinct from MIg but shares the same pathogenetic mechanism (18). This similar immune complex associated glomerular nephritis (ICGN) pattern was also observed in patients (11, 14). Moreover, sparse subendothelial deposits without substructure were observed in our patient, and IgG1 heavy chain and complement were detected by LMD-MS/IF, indicating ICGN rather than fibrinogen deposits alone, which is consistent with the pathogenesis observed in the animal model. Hence, we speculate that CF-GN may exhibit features of either MesPGN or MPGN, with or without IC deposits. The isomeric MIg may exhibit high affinity for Fc fragment accumulation, potentially becoming embedded within the dense central region of the mega-complex due to its crystalline structure. Hence, IgG may have been masked, making it resistant to IF detection. In addition, MS is more sensitive in detecting proteins with substructures, such as those in amyloidosis. Both of these factors could explain the discrepancies between IF and MS detection (39). The diverse pathological characteristics may contribute to the underdiagnosis of CF-GN. Therefore, CF-GN with paraproteinemia should be considered within the spectrum of monoclonal gammopathy of renal significance (MGRS) to better inform treatment strategies. The proposed mechanisms of CF-GN are shown in **Figure 7**.

Cryofibrinogenemia is a treatable and potentially reversible disease (8, 9, 23). The maintenance therapy involves avoiding cold exposure and taking anticoagulants and fibrinolytic agents, whereas the treatment of secondary cryofibrinogenemia involves the management of associated diseases (9, 23). The patient was educated to avoid coldness, but anticoagulants and fibrinolytic agents were not prescribed due to normal coagulation and fibrinolysis levels, and lack of thrombus manifestation. Currently, treatments of kidney diseases are often tailored to the underlying etiology and the severity of renal involvement (11). The presence of CF, immune complex formation, complement activation, and the MPGN pattern suggest that immunosuppressive therapy can be considered to be effective, even in cases with neoplasms (8, 9, 11, 12, 14, 16, 17, 22, 27). Cyclophosphamide was proved to be more effective than FK506, however, relapses were frequently reported (8, 9, 14, 27). Moreover, despite negative results from the exploration of the patient’s hematologic status, continuous hematological supervision and treatment aimed at reducing monoclonal paraprotein levels should be considered accordingly (17).

This study has several important limitations. First, as a single-case investigation, its findings lack generalizability. Second, we were unable to definitively establish a causal relationship between the observed genotype mutations and phenotypic abnormalities. Third, the precise interaction mechanisms between monoclonal immunoglobulin (MIg) and fibrinogen remain to be fully elucidated. Moreover, the short duration with limited stimulation in the animal model may not fully represent kidney injury or facilitate deeper exploration of molecular mechanisms and susceptibility of agents.

In conclusion, cryofibrinogenemia is at risk of being serologically overlooked or pathologically underdiagnosed. MIg has a cryoactivity and a close interaction with fibrinogen, which is responsible for the formation of cryoprecipitates. A thorough MIg detection in elderly patients presenting with CF is essential, and immunosuppressive or monoclonal-targeted therapy should be considered accordingly.

## Data Availability

The data has been uploaded to the Genome Sequence Archive at the National Genomics Data Center, China National Center for Bioinformation/Beijing Institute of Genomics, Chinese Academy of Sciences (accession number: GSA-Human: HRA011861). The data can be viewed here: https://bigd.big.ac.cn/gsa-human/browse/HRA011861.
