# Supplementary material for: Cryofibrinogen-associated glomerulonephritis with paraproteinemia
Source: Front Immunol. 2025 Jul 15;16:1576917. doi: 10.3389/fimmu.2025.1576917 (PMC12305703; doi:10.3389/fimmu.2025.1576917)
Supplement: Supplementary file 1 [file SupplementaryFile1.docx]

**Supplementary materials and methods**

**Immunofluorescence analysis**

Kidney specimens from the patient were snap-frozen, and 3 μm sections were stained with fluorescein isothiocyanate (FITC)-conjugated rabbit antibodies specific for human IgA (F0204, 1:50, DAKO, Denmark), IgG (F0202, 1:50, DAKO, Denmark), IgM (F0203, 1:50, DAKO, Denmark), C3c (F0201, 1:50, DAKO, Denmark), C1q (F0254, 1:50, DAKO, Denmark), κ (F0198, 1:50, DAKO, Denmark), λ (F0199, 1:50, DAKO, Denmark). In addition, Kidney specimens from the mouse model were also snap-frozen, and 3 μm sections were stained with Alexa Fluor (AF) 594-conjugated goat anti-mouse IgG (ab150116, 1:100, Abcam, USA), AF647-conjugated goat anti-mouse IgM (ab150123, 1:100, Abcam, USA), and FITC-conjugated goat anti-mouse IgA (ab97234, 1:100, Abcam, USA) by IF method.

Frozen tissues were incubated by primary antibodies above under 4°C overnight. The fluorescent sections were observed under Olympus BX51 immunofluorescence microscope.

The paraffin embedded tissues were rehydrated and antigen retrieved by Tris-EDTA PH9.0 (MVS-0098, Maxim Corporation, China) or citric acid PH6.0 (MVS-0100, Maxim Corporation, China), and proteinase K digestion (PKT-0011, Maxim Corporation, China). Followed by primary antibodies above under 4°C overnight.

**Immunohistochemical analysis**

Following rehydration, antigen was unmasked using high pressure method for 5 min by antigen retrieval Tris-EDTA PH9.0 (MVS-0098, Maxim Corporation, China). Followed by 10 min endogenous peroxidase block by hydrogen peroxide blocking reagent (ab64218, Abcam, Shanghai, China) and 30 min nonspecific binding block by PBS containing 3% BSA, sections were incubated with the primary antibodies, rat anti-mouse C5b-9 (sc-66190, 1:50, Santa Cruz, USA) and mouse anti-human CD68 (Kit-0026, Maxim Corporation, China) under 4°C overnight. An isotype-matched irrelevant antibody was used as a negative control. Next, slides were incubated with MaxVisionTM HRP-Polymer anti-Mouse/Rabbit IHC Kit (KIT-5010, Maxim Corporation, China) under 37°C for half an hour. Peroxidase activity was visualized with diaminobenzidine (KGP1045-100, KeyGEN BioTECH, Jiangsu, China). The nucleus were stained with hematoxylin followed by dehydration and mounting in neutral balsam (ZLI-9555, ZSGB-BIO, Beijing, China). Slides were observed under an Olympus BX51 light microscope.

**Electron microscopy**

Kidney biopsy tissues and organ tissues from mouse models were sliced into small pieces (approximately 2 mm^3^), fixed in 2.5% glutaraldehyde, post-fixed in 1% osmium tetroxide, and embedded into Epon 812 resin. Ultrathin sections were stained with uranyl acetate and lead citrate and examined using a transmission electron microscope (JEM-1230, JEOL, Tokyo, Japan). The cryoprecipitates were centrifuged, embedded into 2.5% agarose, and fixed in 2.5% glutaraldehyde overnight. The subsequent procedures were the same as tissues.

**Immuno electron microscopy (IEM)**

Tissues were transferred to nickel grids and incubated in 1% H2O2 for 10min, followed by incubation with bovine serum albumin for 30min, polyclonal rabbit anti-human fibrinogen (1:50, Sigma-Aldrich, Saint Louis, USA) incubation overnight at 4°C, and gold-labeled second antibody (25109, 1：20, EMS, USA) incubation for 60min. All of the samples detected by EM were observed under a transmission electron microscope (JOEL-1400, Japan).

**Mass spectrometry (MS)**

Paraffin-embedded kidney tissue from the patient was microdissected using the Leica dissector (Leica DM 600 B). Microdissected tissues were collected into PCR tube caps containing 20 µL Tris/ EDTA/0.002% Zwittergent 3–16 bufer and digested with tryptic peptides (Promega Corporation, Madison, WI) overnight at 37°C. After desalting using ZipTip C18 micro columns (Millipore, Billerica, MA), samples were analyzed using an EASY-nLC 1200 liquid chromatography system with Reprosil-Pur 120 C18 columns for online separation coupled to a Q-Exactive HF-X Orbitrap mass spectrometer (Termo Fisher Scientifc GmbH, Bremen, Germany) using a mobile phase consisted of bufer A (0.1% formic acid, FA) and a 120-min gradient of increasing concentrations of bufer B (0.1% FA, 80% acetonitrile) at a flow rate of 600 nL per minute. The linear gradient method began at 4% buffer B, and the concentration was increased to 7% for 1min, with subsequent increases to 25% (95min), 40% (111min), 100% (116min), and a hold for 4min. Mass spectra were acquired under data-dependent acquisition (DDA) mode, consisting of full MS1 scans (m/z range, 350–2000; resolution: 60,000) followed by MS2 scans of the top 20 parent ions (resolution of 15,000 AGC target to 5e4 , maximum injection time of 45 ms). The protein probability scores were evaluated in Scaffold (Proteome Software, Portland, Oregon, USA).

**Genetic analysis by next‑generation sequencing, PCR and quantitative RT-PCR**

Genomic DNA from peripheral blood of the patient was extracted and fragmented using the QIAamp DNA Mini Kit (Qiagen, Shanghai, China). A DNA Sample Prep Reagent Set (MyGenostics, Beijing, China) was used for the preparation of standard libraries. A thrombus and hemostasia related gene panel, including fibrinogen, was analyzed by next‑generation sequencing (NGS). The amplified DNA was captured using the GenCap capture kit (MyGenostics Inc, Beijing, China). The subsequent bioinformatics analysis was performed using cutadaptor software (<http://code.google.com/p/cutadapt/>), Sentieon software (<https://www.sentieon.com/>), ANNOVAR software (<http://annovar.openbioinformatics.org/en/latest/>), and CNVkit software (<https://cnvkit.readthedocs.io/en/stable/>). All mutations identified by DNBSEQ-T7 sequencing were confirmed by Sanger sequencing. Genomic DNA from all available family members were obtained for Sanger sequencing. The effect of gene mutations on protein structure and function was predicted by SIFT, PolyPhen-2, MutationTaster, and GERP++.

PCR amplification was performed using FGB-specific exon-spanning primers (spanning exons 1–7). The PCR products were then separated on a 1% agarose gel and visualized using a gel imaging system.

The primer sequences were as follows:

FGB-cDNA-F：AGTCCCAAGGTGTCAACGA

FGB-cDNA-R：CCGTCATTGTCTCTGTCATACG


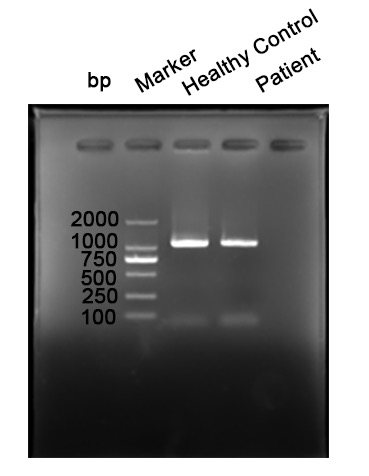


As shown in the image above, the PCR product bands of both the healthy control and the patient were 1156 bp in size, which indicates that the mutation in intron 4 does not affect the splicing of FGB.

Total RNA was extracted from peripheral blood using an RNA purification kit (Servicebio, Wuhan China). cDNA was synthesized using a Primescript™ RT reagent kit (TaKaRa, Kusatsu, Japan). Quantitative real-time PCR (qRT-PCR) of exons of fibrinogen mRNA (50-150bp around and 500bp downstream of the mutation site) was performed on an Applied Biosystems QuantStudio 5 Real-Time PCR System using TB Green® Premix Ex Taq™ (Takara). The relative abundance of fibrinogen mRNA was normalized to that of GAPDH using the comparative cycle threshold method (2−ΔΔCT). Each PCR experiment was performed in triplicate and repeated independently at least three times. Gene mutation-related exons of the amplified cDNA were confirmed by Sanger sequencing.

The primer sequences were as follows:

FGB-F AGGCGGGCGGTCACAAGG

FGB-R GTGGCAGAGTGGTGGCTTTTCC

FGG-F GTGGAACTGGAAGACTGGAATGG

FGG-R ATATGTTAGGCGGTACTTGTCAGC

FGA-F GCAATGACTACCTCCACTTACTAACC

FGA-R CCTTCAGCCTCAGAGCCTACC

**Animal Model**

Eight male C57BL/6 mice, aged 6-8 weeks and weighing 16-24g, were purchased from Shanghai Sippe-Bk Lab Animal Corporation. Four mice were injected intraperitoneally with precipitates once each month (five months in total). The precipitates were dissolved in 1 ml of 0.9% sodium chloride. Two mice were injected with 300ul for each and they were categorized as a high-dose group, whereas the other two mice belonged to the low-dose group with 200ul injection for each. Low-dose1 was sacrificed after the third round of injection, and low-dose2 was sacrificed after the fourth round. The two mice (high-dose 1 and 2) were sacrificed together at the last round of injection. Urine was collected each month and examined for urinary protein-to-creatinine ratio (UPCR). Blood was obtained twice (before injection and at the spot of sacrifice) to test ALB and Scr. Six organs including the brain, lung, heart, liver, kidney and intestine, and skin were obtained and processed for LM and EM. Twenty glomeruli were assessed in each kidney and the proportion of mesangial matrix area per glomerulus was analyzed by using Image-Pro Plus 6.0 software. Four mice were set as negative controls.

**Analysis of urine total protein, serum creatinine and albumin in mouse model**

A mouse ELISA kit was used to detect urine total protein (C035-2-1, Nanjing Jiancheng Bioengineering Institute, Nanjing, JS, China). Urinary creatinine levels were measured by a creatinine assay kit (DICT-500, BioAssay Systems, Hayward, CA, USA). Serum creatinine levels were measured by a kit (C011-2-1, Nanjing Jiancheng Bioengineering Institute, China). Serum albumin levels were measured by a kit (A028-2-1, Nanjing Jiancheng Bioengineering Institute, China). Proteinuria was defined as the ratio of urine total protein/creatinine ratios (UPCR, mg/g).

**Cell culture**

Human mesangial cells (HMCs) were cultured in DMEM (Gibco, Grand Island, NY), supplemented with 10% fetal bovine serum (FBS, Biological Industries, Israel), as well as 100 U/ml penicillin and 100 mg/ml streptomycin (Beyotime, Shanghai, China) in a cell incubator with 5% CO2 at 37°C. HMCs were treated with 1.29 g/l cryoprecipitates, approximately 3g/l purified IgG from the patient and other individuals, and vehicle cotrol (diluted with DMEM) for 24 h to assess cell proliferation, fibrinogen/MIg absorption, and morphological features by EM. Cell proliferation was assessed using the BeyoClickTM 594 EdU kit (Beyotime, Shanghai, China) and detected with Harmony 4.8 imaging and analysis software (PerkinElmer). Fibrinogen/MIg detection in cells was processed using Cytospin 4 (Thermo) and double-stained with propidium iodide (P4170, Sigma-Aldrich, USA) and rabbit anti-human IgG (F0202, DAKO, USA) by IF. The proportion of proliferative cells and fibrinogen/IgG-positive cells was analyzed by Image-Pro Plus 6.0 software. EM of mesangial cells was performed according to the protocol above.

**IgG λ purification and molecular docking**

The patient’s serum was dissolved in double-volume of PBS buffer and then incubated with 2 mL of Protein A beads at 4℃ for 4 hours. After centrifugation for 10 min, the pellet was loaded onto a gravity column. The fraction was collected after being eluted with cold PBS containing 10 mM Glycine. Samples of approximately 1 mL centrifuged at 4000 rpm at 4℃ for three times, were applied to a Superdex 200 HR 10/30 column(Amersham Biosciences), and the peak fraction was collected for further experiments. Molecular docking of fibrinogen and IgG was conducted using the DOCK SERVER(<https://zdock.wenglab.org/>). The interaction interface was visualized by Chimera in Cartoon (A) and surface (right) model. The residues involved in interface were shown in stick model colored yellow.
